# Checking Perspiration

**Published:** 1882-04

**Authors:** 


### CHECKING PERSPIRATION.

A BOSTON merchant, in "lending a hand" on board of one of his ships on a windy day, found himself at the end of an hour and a half pretty well exhausted and perspiring freely He sat down to rest. The cool wind from the sea was delightful, and engaging in conversation, time passed faster than he was aware of. In attempting to rise, he found he was unable to do so without assistance. He was taken home and put to bed, where he remained two years; and for a long time afterwards, could only hobble about with the aid of a crutch. Less exposures than this have, in constitutions not so vigorous, resulted in inflammation of the lungs, "pneumonia," ending in death in less than a week, or causing tedious rheumatisms, to be a source of torture for a lifetime. Multitudes of lives would be saved every year, and an incalculable amount of human suffering would be prevented, if parents would begin to explain to their children at the age of three or four years, the danger which attends cooling off too quickly after exercise, and the importance of not standing still after exercise, or work, or play, or of remaining exposed to a wind, or of sitting at open window or door, or of pulling off any garment, even the hat or bonnet, while in a heat. It should be remembered by all, that a cold never comes without a cause, and that in four times out of five, it is the result of leaving off exercise too suddenly or of remaining still in the wind, or in a cooler atmosphere than that in which the exercise has been taken.

The colder the weather the more need is there, in coming into the house, to keep on all the clothing, except India-rubber or damp shoes, for several minutes afterwards. Very few rooms are heated higher than sixty-five degrees when the thermometer is within twenty degrees of zero, while the temperature of the body is always at ninety-eight, in health; so that if a man comes into a room which is thirty degrees colder than his body, he will rapidly cool off, too much so often, even if the external clothing is not removed.

It is not necessary that the perspiration be visible; any exercise which excites the circulation beyond what is natural, causes a proportional increase of perspiration, the sudden checking of which induces dangerous diseases and certain death every day

### QUENCHING THIRST.

Nearly a hundred years ago, Dr. Lind suggested to Captain Kennedy, that thirst might be quenched at sea, by dipping the clothing in salt water, and putting it on without wringing. Subsequently the Captain on being cast away, had an opportunity of making the experiment. With great difficulty, he succeeded in persuading a part of the men to follow his example, and they all survived ; while the four who refused and drank salt water, became delirious, and died. In addition to putting on the clothes while wet, night and morning, they may be wetted while on, two or three times during the day. Captain K. goes on to say, "after these operations, we uniformly found that the violent drought went off, and the parched tongue was cured in a few minutes after bathing and washing our clothes, while we found ourselves as much refreshed, as if we had received some actual nourishment."

The bare possibility of the truth of the statement, makes it a humanity for any paper to give it a wide publicity, since there are few readers in any hundred, who may not go to sea and be shipwrecked.

We personally know that wading in water quenches thirst, and very few readers can remember being thirsty while bathing at the sea shore, or while swimming in our rivers. When the fearful horrors of dying with thirst are remembered, and the more fearful madness which is the certain result of drinking sea water to allay thirst, it is certainly well to encourage individual experiment in this direction, and solicit an authenticated report of the same.

---

### "DRINKING" AND DEATH

Of soul, body and estate, how certain, soon and swift the sequence. R. M. H ———., who for a full quarter of a century has been an untiring minister of good to the sick and poor among us says that, half of all the city charities are swallowed up in removing the ills occasioned by intemperance.

We believe that if the sale of liquor of every name could be prevented, from sunset to sunrise, of every day, and for the whole of Sunday, it would be a direct annual saving of a million of dollars to the city treasury ; and that crime, from petty theft to deliberate murder, would be prevented every year, aye, every month! enough to make an angel weep for joy. All honor to the men who are giving their time, their influence, their money, and more than all, their personal labor, to break up "The Sunday Liquor Traffic." It is a nobler effort than to win a battle, or found an empire, for it will be if successful, the saving of men enough to people an empire, every year!

